# Comparative Genomics Uncovers the Genetic Diversity and Characters of *Veillonella atypica* and Provides Insights Into Its Potential Applications

**DOI:** 10.3389/fmicb.2020.01219

**Published:** 2020-06-23

**Authors:** Maozhen Han, Gang Liu, Yajun Chen, Dong Wang, Yan Zhang

**Affiliations:** ^1^School of Life Sciences, Anhui Medical University, Hefei, China; ^2^School of Life Sciences, Hefei Normal University, Hefei, China

**Keywords:** *Veillonella atypica*, pan-genome, CAZymes, metabolic pathways, CAS-Type IIIA

## Abstract

*Veillonella atypica* is a bacterium that is present in the gut and the oral cavity of mammals and plays diverse roles in different niches. A recent study demonstrated that *Veillonella* is highly associated with marathon running and approved that *V. atypica* gavage improves treadmill run time in mice, revealing that *V. atypica* has a high biotechnological potential in improving athlete performance. However, a comprehensive analysis of the genetic diversity, function traits, and genome editing method of *V. atypica* remains elusive. In the present study, we conducted a systemically comparative analysis of the genetic datasets of nine *V. atypica* strains. The pan-genome of *V. atypica* consisted of 2,065 homologous clusters and exhibited an open pan-genome structure. A phylogenetic analysis of *V. atypica* with two different categories revealed that *V. atypica* OK5 was the most distant from the other eight *V. atypica* strains. A total of 43 orthologous genes were identified as CAZyme genes and grouped into 23 CAZyme families. The CAZyme components derived from accessory clusters contributed to the differences in the ability of the nine *V. atypica* strains to utilize carbohydrates. An integrated analysis of the metabolic pathways of *V. atypica* suggested that *V. atypica* strains harbored vancomycin resistance and were involved in several biosynthesis pathways of secondary metabolites. The *V. atypica* strains harbored four main *Cas* proteins, namely, CAS-Type IIIA, CAS-Type IIA, CAS-Type IIC, and CAS-Type IIID. This pilot study provides an in-depth understanding of and a fundamental knowledge about the biology of *V. atypica* that allow the possibility to increase the biotechnological potential of this bacterium.

## Introduction

*Veillonella atypica*, which belongs to family *Veillonellaceae* and genus *Veillonella*, is a Gram-negative bacterium and anaerobic coccus. To date, species of genus *Veillonella*, including *V. atypica*, but with the exception of *Veillonella seminalis* (Aujoulat et al., 2014), are well known for their lactate fermenting abilities, allowing them to utilize lactate and transform it to propionate and acetate (Kolenbrander, 2006). The strains of *V. atypica* can be found in the intestines and the oral mucosa of mammals (Beighton et al., 2008; Vesth et al., 2013) and play diverse roles in different niches. For example, the coaggregation properties of *Veillonella* spp. of the human oral cavity affect the colonization site and the ecology of oral microbial communities (Hughes et al., 1988). Moreover, oral *Veillonella* spp., including *V. atypica*, together with *Streptococcus* spp., are known as early colonizers in oral biofilm formation and affect the development of dental plaques (Mashima and Nakazawa, 2015). A case report stated that *V. atypica*, along with *Actinomyces odontolyticus*, led to pulmonary infection in a 65-year-old, male immunocompetent patient with dental caries (Crisafulli et al., 2019). Besides that, metal reduction by microbes has attracted increased attention (Lloyd, 2003), and numerous microorganisms with the ability to reduce metals have been isolated, identified, and studied (Zhang et al., 2019a, b; Wang et al., 2020). As a metal-reducing bacterium, *V. atypica* has been identified as a selenium-respiring bacterium and possesses the ability to transform biogenic selenite (Se) (Nancharaiah and Lens, 2015). *V. atypica* reduces Se(IV) by using hydrogen as electron donors and involves a metal-reducing mechanism different from that of *Geobacter sulfurreducens* and *Shewanella oneidensis* (Pearce et al., 2009). Following the effective detoxification of Cr(VI) *via* a sulfur-based mixotrophic bio-reduction process (Zhang et al., 2020), bioengineering of *V. atypica* strains will increase their potential applications as selenium-respiring bacterium with an important role in the selenium cycle. However, the genes and/or proteins associated with this mechanism remain elusive. Understanding the functional traits of genes and/or proteins of *V. atypica* will be beneficial in increasing the ability of this species to transform biogenic selenite. Additionally, a study found that the relative abundance of gut *Veillonella* is significantly associated with marathon running and *V. atypica* gavage improves treadmill run time in mice (Scheiman et al., 2019). The mechanism involves crossing of serum lactate from the epithelial barrier into the gut lumen, and then the gut *V. atypica* transforms lactate to acetate and propionate, which is sufficient to improve treadmill run time (Scheiman et al., 2019). Therefore, understanding the genetic diversity and the functional characteristics of *V. atypica* will expand its biotechnological applications in environmental restoration and exercise performance.

To date, nine *V. atypica* strains, namely, *V. atypica* KON, *V. atypica* CMW7756B, *V. atypica* ACS-049-V-Sch6, *V. atypica* ACS-134-V-Col7a, *V. atypica* KON ATCC 17744, *V. atypica* NCTC11830, *V. atypica* AF36-15BH, *V. atypica* KHUD_V1, and *V. atypica* OK5, have been sequenced and their genome sequences can be available in public database. The genome of strain *V. atypica* OK5 was the first to be completely sequenced among the *V. atypica* strains. This strain was isolated from a human saliva sample to reveal the genetic transformability among the species of *Veillonella* genus. Based on the complete genome sequence of *V. atypica* OK5, a genetic study provided an in-depth understanding of the ecology of human oral biofilms (Zhou et al., 2015). The genome sequences of the other eight strains are incomplete. A study of the genetic diversity of genus *Veillonella* and 137 prokaryotic genomes, including eight *Veillonella* spp., suggested that the genus *Veillonella* is relatively homogeneous (Vesth et al., 2013). However, the genetic diversity and the functional traits of *V. atypica*, especially concerning the composition of carbohydrate-active enzymes (known as CAZy enzymes or CAZymes) and CRISPR–Cas system, which is widely used in editing the genetic elements of microbiota (Alkhnbashi et al., 2019; Horvath and Barrangou, 2010), remain unclear. Hence, a comprehensive analysis of the characteristics of *V. atypica* is warranted.

In this study, we collected the available genomic datasets of nine *V. atypica* strains and conducted a comparative analysis to investigate the strains’ genetic diversity, functional traits, including CAZyme, Cluster of Orthologous Group (COG), Gene Ontology (GO) function, and metabolic pathways, and CRISPR–Cas composition. The results revealed differences in the genetic features of these strains and demonstrated that *V. atypica* exhibited an open pan-genome structure. The evolutionary trees of nine *V. atypica* strains constructed with two different strategies showed that the nine *V. atypica* strains can be divided into two clusters, and *V. atypica* OK5 may not the suitable representative strain for *V. atypica*. The differences in the ability of the strains to utilize carbohydrates were derived from the CAZyme component of accessory clusters. Several proteins of *V. atypica* were annotated to the Kyoto Encyclopedia of Genes and Genomes (KEGG) pathways involved in antibiotic resistance and biosynthesis of secondary metabolites. The *V. atypica* strains harbored four main *Cas* proteins, namely, CAS-Type IIIA, CAS-Type IIA, CAS-Type IIC, and CAS-Type IIID.

## Results and Discussion

### Pan-Genome Construction and Analysis

In general, the use of complete genomes is recommended for estimating the genetic diversity in pan-genome analysis, and the mixture of complete and draft genomes is also allowed in pan-genome analysis (Veras et al., 2018). For example, the genetic diversity of *Corallococcus* was evaluated on the basis of two complete genomes and 21 draft genomes of *Corallococcus* strains *via* pan-genome analysis (Livingstone et al., 2018), and the 11 genomes (five complete and six draft genomes) were used to obtain a first approximation of the *Piscirickettsia salmonis* pan-genome (Bravo and Martinez, 2016). Therefore, in this present study, to conduct the comparative analysis of species *V. atypica*, nine available genomic datasets, including complete and draft genomes, and their corresponding protein datasets for *V. atypica* strains were collected and downloaded from National Center for Biotechnology Information (NCBI). Except for *V. atypica* OK5, the sequenced genomes of the *V. atypica* strains were mainly assembled at contig and scaffold levels, and the number of scaffolds ranged from 1 to 94. The assembled genome of *V. atypica* ranged from 1.99 to 2.19 Mb, whereas the protein number of *V. atypica* ranged from 1,781 to 1,973 (Table 1). The results showed that the nine *V. atypica* strains were mainly isolated from the oral cavity, vagina, and feces of *Homo sapiens* (Table 1). It is important to note that although this study is limited in that the pan-genome analysis was performed with a restricted quantity of fully sequenced genomes, the genetic diversity and the characteristics of *V. atypica* were explored with mainstream databases and tools of pan-genome analysis, which was used to evaluate the genetic diversity and the functional traits of *Aeromonas* (Zhong et al., 2019) and *Shewanella* (Zhong et al., 2018) *via* pan-genome analysis. This pilot study is an important step toward understanding the functional traits and the functional variants of *V. atypica*.

**TABLE 1:** Genomic datasets of the nine *Veillonella atypica* strains used in this study. The genomic features of the nine *V. atypica* strains were collected from National Center for Biotechnology Information.

Strain	Size (Mb)	GC%	Scaffolds	Proteins	Level	Isolation source	Host
*V. atypica* KON	1.99716	39	40	1903	Scaffold	Oral cavity	*Homo sapiens*
*V. atypica* CMW7756B	2.09885	38.9	94	1973	Scaffold	Vagina	*Homo sapiens*
*V. atypica* ACS-049-V-Sch6	2.05387	39	63	1840	Contig	Oral cavity	*Homo sapiens*
*V. atypica* ACS-134-V-Col7a	2.15191	39	70	1903	Contig	Oral cavity	*Homo sapiens*
*V. atypica* KON ATCC 17744	2.03741	39	11	1832	Contig	Oral cavity	*Homo sapiens*
*V. atypica* NCTC11830	2.10794	39	8	1957	Contig	Oral cavity	*Homo sapiens*
*V. atypica* AF36-15BH	1.99286	38.9	24	1781	Scaffold	Feces	*Homo sapiens*
*V. atypica* KHUD_V1	2.18929	39	54	1969	Scaffold	Oral cavity	*Homo sapiens*
*V. atypica* OK5	2.07195	39.1	1	1790	Complete Genome	Oral cavity	*Homo sapiens*

**FIGURE 1:**
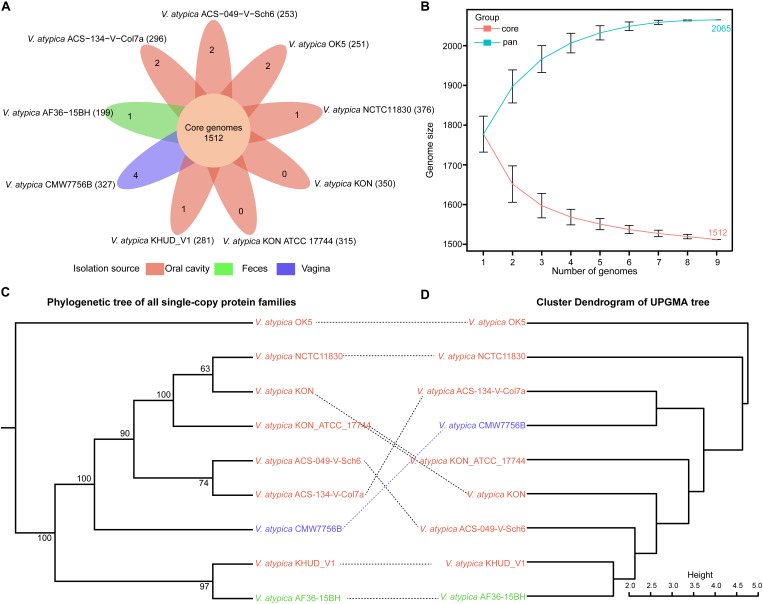
Genetic diversity in *Veillonella atypica* strains. **(A)** Core, accessory, and specific gene families of the nine *V. atypica* strains. The number of core genomes shared by all strains is in the center (1,512). The number of non-overlapping portions of each oval represents the size of specific families (unique genes). The number of accessory families of each strain is shown in parentheses after the strain name. **(B)** The size of pan-genome (top) and core genome (bottom) shared by different strains, respectively. Phylogenetic relationship of nine *V. atypica* strains. **(C)** Phylogenetic tree based on all single-copy protein sequences. **(D)** The UPGMA tree of Manhattan distance based on the pan-genome composition of the nine *V. atypica* strains.

To characterize the differences in genomic features among these nine *V. atypica* strains, the orthologs were identified from 16,948 high-quality proteins of *V. atypica*. A total of 2,065 homologous clusters were identified from *V. atypica* strains. Among these homologous clusters, 1,512 homologous clusters (73.22%) were found in all the nine *V. atypica* strains and accordingly identified as the core genome of *V. atypica* (Figure 1A). In addition, 1,468 homologous clusters from the core genome were identified as single-copy core families. The number of accessory families of *V. atypica* ranged from 199 to 376 (Figure 1A), whereas 13 homologous clusters were identified as specific families (unique genes) for the nine *V. atypica* strains, the number of specific families of which ranged from 0 to 4. Interestingly, no specific families were found in the strains of *V. atypica* KON and *V. atypica* KON ATCC 17744. Additionally, the number of genes in the specific families of the other *V. atypica* strains were all more than two, suggesting that these multi-copy specific genes may play an important role in the adaptation of the *V. atypica* strains to specific niches.

Moreover, the accumulation curve of homologous clusters has just reached the plateau stage, which suggested that *V. atypica* exhibited an open pan-genome structure (Figure 1B). With the emergence of new *V. atypica* strains, the pan-genome size of *V. atypica* tended to increase gradually, whereas the core genome size of *V. atypica* tended to decrease progressively. Moreover, the pan-genome and the core-genome sizes of *V. atypica* were estimated to be 2,065 and 1,512 non-redundant genes within the nine *V. atypica* strains, respectively (Figure 1B). The distribution of the core, accessory, and specific genes revealed that the genomes of the *V. atypica* strains were remarkably diverse.

### Phylogenetic Analysis of *V. atypica* Strains

To compare the similarity and the distance of the *V. atypica* strains and gain insights into the evolutionary relationship of these nine *V. atypica* strains, two strategies were applied to construct the phylogenetic trees and explore the phylogenetic relationships among the nine *V. atypica* strains. One strategy was based on the protein sequences of concatenated alignments of 1,468 single-copy core genes shared by the nine *V. atypica* strains (Figure 1C). This strategy can eliminate the effects of genome size and sequencing quality. The other strategy was based on the Manhattan distance between the nine *V. atypica* strains. The distance was calculated on the basis of the absence or the presence of each protein homolog (Figure 1D). Although the information from NCBI BioSample and the List of Prokaryotic names with Standing in Nomenclature (LPSN)^1^ revealed that *V. atypica* KON, *V. atypica* KON ATCC 17744, and *V. atypica* NCTC11830 are the same strain and the result of the phylogenetic tree of 16S rRNA showed that these three strains are in the same cluster (Supplementary Figure S1), the result in Figure 1D showed that *V. atypica* KON, *V. atypica* NCTC11830, and *V. atypica* KON ATCC 17744 are not in a same cluster. These results suggested that *V. atypica* KON, *V. atypica* KON ATCC 17744, and *V. atypica* NCTC11830 are different substrains of *V. atypica* KON. We speculated that the genetic variations of these three substrains contributed to their remarkable differences. For example, the number of 16S rRNA gene copies of these substrains ranged from 1 to 4. One 16S rRNA gene copy was presented in *V. atypica* KON, four 16S rRNA gene copies were presented in *V. atypica* KON ATCC 17744, and three 16S rRNA gene copies were presented in *V. atypica* NCTC11830. The results of the similarity analysis among the genomes of *V. atypica* KON, *V. atypica* NCTC11830, and *V. atypica* KON ATCC 17744 showed that the symmetric identity between *V. atypica* KON ATCC 17744 and *V. atypica* KON is 98.91%, while the symmetric identity between *V. atypica* KON ATCC 17744 and *V. atypica* NCTC11830 is 97.51%. In these two trees, *V. atypica* KHUD_V1 showed a closer evolutionary relationship with *V. atypica* AF36-15BH than with the other strains, although these two strains were isolated from different sources (oral cavity and feces). A previous study reported that *V. atypica* OK5 was isolated from a human saliva sample and the first transformable strain in *Veillonella* genus (Zhou et al., 2017). Another study sequenced the complete genome and successfully established a counter-selectable markerless mutagenesis system for the strain *V. atypica* OK5; these advances made the strain *V. atypica* OK5 an important and preferred strain in genetic studies of *Veillonella* genus (Zhou et al., 2015). However, the two different phylogenetic trees in the present study revealed that the evolutionary relationship of *V. atypica* OK5 was farther than previously thought from that of the remaining eight *V. atypica* strains (Figures 1C,D). This result suggested that *V. atypica* OK5 may not be a suitable representative strain of *V. atypica*. Hence, additional strains should be re-selected for sequencing and model construction to facilitate future studies on *Veillonella* genus. Additionally, although *V. atypica* NCTC11830, *V. atypica* KON, *V. atypica* KON ATCC 17744, *V. atypica* ACS-049-V-Sch6, *V. atypica* ACS-134-V-Col7a, and *V. atypica* CMW7756B showed closer phylogenetic relationships with one another, their evolutionary relationships remained intricate (Figures 1C,D). This observation suggested that these strains were dissimilar. Moreover, these strains contained more accessory families than *V. atypica* KHUD_V1, *V. atypica* AF36-15BH, and *V. atypica* OK5, with the exception of *V. atypica* ACS-049-V-Sch6. Furthermore, the numbers of accessory families of these six strains were diverse. Hence, we speculated that the gain and the loss of different genes from different strains contributed to the large differences in the genetic compositions of *V. atypica* strains and led to evolutionary divergence among *V. atypica* strains.

### Identification of CAZymes for *V. atypica* Strains

**FIGURE 2:**
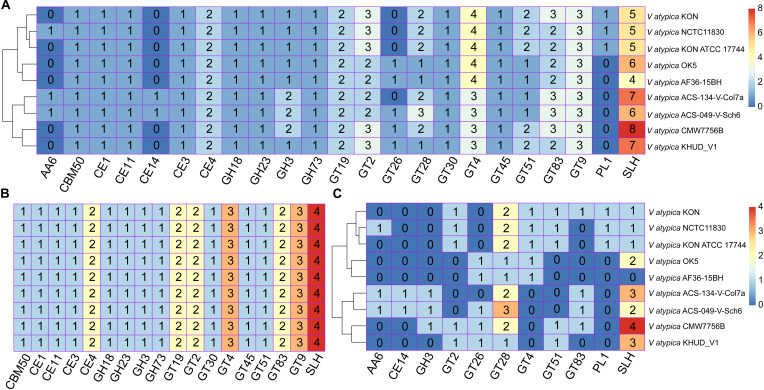
Distribution of CAZymes in the nine *Veillonella atypica* strains. **(A)** Distribution of CAZymes of pan-genome in each *V. atypica* strain. **(B)** Distribution of CAZymes of pan-genome in each *V. atypica* strain grouped by core clusters. **(C)** Distribution of CAZymes of pan-genome in each *V. atypica* strain grouped by accessory clusters. The numbers shown in subgraphs represent the orthologous genes assigned by CAZymes. GT, glycosyltransferase; GH, glycoside hydrolase; CE, carbohydrate esterase; CBM, carbohydrate-binding molecule; AA, auxiliary activities; PL, polysaccharide lyases; SLH, S-layer homology.

Previous studies have reported that the members of *Veillonella* are generally unable to ferment carbohydrates but rather grow well on media containing lactate, pyruvate, malate, or fumarate under anaerobic conditions (Kolenbrander, 2006). The members of *Veillonella* have been suggested to exhibit unusual metabolism of carbohydrates, but this type of metabolism has not been clarified in the *V. atypica* strains. Hence, in the present study, 2,065 homologous clusters of *V. atypica* were systemically identified against the CAZy database to obtain a comprehensive understanding of the catalytic ability of carbohydrate of *V. atypica* (Yin et al., 2012). A total of 43 orthologous genes were identified and grouped into 23 CAZyme families (Figure 2A). Among these 43 orthologous genes, 29 and 14 orthologous genes belonged to accessory clusters and core clusters and categorized into 11 and 18 CAZyme families (Figures 2B,C), respectively. The number in Figure 2 represents the number of genes classified into each CAZyme family for each *V. atypica* strain and shows the distribution of CAZyme families in the nine *V. atypica* strains. It was also noted that the 14 orthologous genes belonging to the core clusters all belonged to the single-copy gene clusters and the distribution of CAZyme derived from the core clusters are same in the *V. atypica* strains (Figure 2B). We observed that the CAZymes derived from the core clusters were mainly divided into glycosyltransferase (GT), glycoside hydrolase (GH), and carbohydrate esterase (CE). Previous studies have reported that the primary function of GTs is to catalyze glycoside synthesis, the substrates of which are various sugar-1-phosphate derivatives (Lairson et al., 2008). During the process of reaction, GT utilizes activated sugar donors and catalyzes glycosyl group transfer to appropriate acceptors (Martinez-Fleites et al., 2006). In the present study, eight kinds of GTs derived from the core clusters were found in the nine *V. atypica* strains, namely, GT19, GT2, GT30, GT4, GT45, GT51, GT83, and GT9 (Figure 2B). Among these GT families, GT2 and GT4 families are comprised of chitin synthase, cellulose synthase, α-glucosyltransferase, etc. (Bohra et al., 2019), and GT4 is one of the largest families and contains several enzymes that contribute to lipopolysaccharide synthesis and antibiotic avilamycin A synthesis (Martinez-Fleites et al., 2006). GT9 and GT19 are heptosyltransferase (lipopolysaccharide N-acetylglucosaminyltransferase) and lipid-A-disaccharide synthase, respectively, and they contribute to the synthesis of lipopolysaccharide (Qi et al., 2019). In the present study, the numbers of GT2, GT4, GT9, and GT19 were pre-dominant in the nine *V. atypica* strains. Owing to the presence of CAZyme families, *V. atypica* has an unusual preference for organic acid carbon sources and plays a crucial role in the removal of toxic metabolites from biofilm communities (Delwiche et al., 1985; Knapp et al., 2017).

Additionally, the differences in CAZymes among the nine *V. atypica* strains were attributed to the CAZyme components derived from the accessory clusters (Figure 2C). For example, *V. atypica* NCTC11830, *V. atypica* ACS-134-V-Col7a, and *V. atypica* ACS-049-V-Sch6 were found to harbor one orthologous gene belonging to AA6, which is 1,4-benzoquinone reductase and involved in the biodegradation of aromatic compounds (Luo et al., 2018). *V. atypica* ACS-134-V-Col7a and *V. atypica* ACS-049-V-Sch6 harbored one orthologous gene belonging to CE14, whereas *V. atypica* KON, *V. atypica* ACS-134-V-Col7a, *V. atypica* ACS-049-V-Sch6, and *V. atypica* CMW7756B harbored one orthologous gene belonging to GT83 (Figure 2C). The distributions of CAZymes in the nine *V. atypica* strains were different, suggesting the remarkable differences in the ability of each *V. atypica* strain in carbohydrate metabolism.

### Functional Traits of *V. atypica* Strains

**FIGURE 3:**
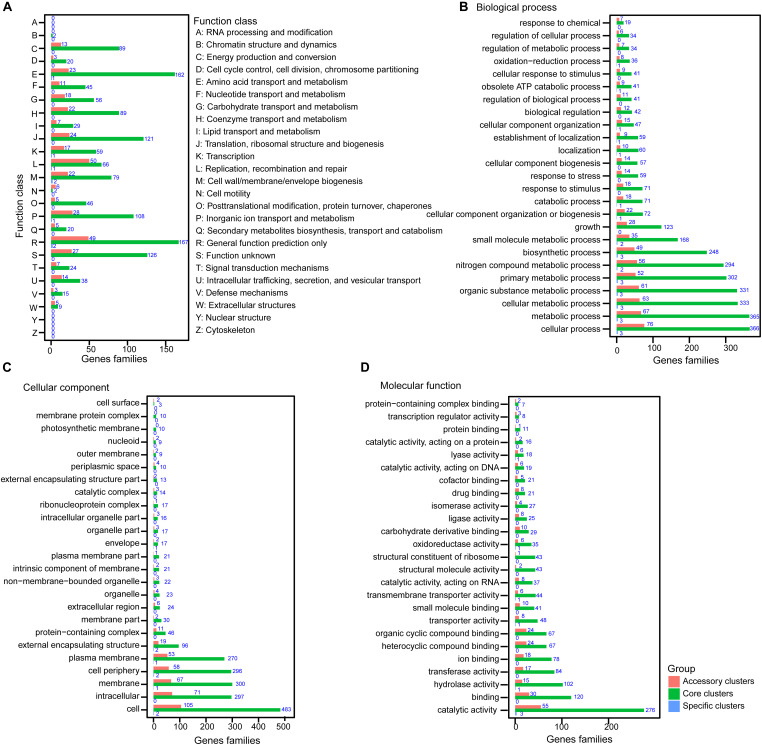
Cluster of Orthologous Group (COG) and Gene Ontology (GO) annotations of gene families for the pan-genome of *Veillonella atypica*. **(A)** Distribution of 25 functional categories according to COG annotation for *V. atypica*. Distribution of GO annotation of pan-genome in **(B)** biological process, **(C)** cellular component, and **(D)** molecular function for *V. atypica*. The pink, green, and wathet blue bars of each subgraph represent the accessory, core, and specific clusters, respectively.

The pan-genome of *V. atypica* was profiled against COG to gain insight into the functional composition of *V. atypica* and characterize its functional traits for the accessory, core, and specific clusters (Figure 3A). The orthologous clusters of the pan-genome were mainly dominant in “general function” (R, 218, 12.54%), “amino acid transport and metabolism” (E, 186, 10.7%), and “translation, ribosomal structure, and biogenesis” (J, 145, 8.34%). The functions of the accessory clusters were mainly enriched in “replication, recombination, and repair” (L, 50, 13.93%), “general function” (R, 49, 13.65%), “inorganic ion transport and metabolism” (P, 28, 7.8%), “amino acid transport and metabolism” (E, 23, 6.41%), “translation, ribosomal structure, and biogenesis” (J, 24, 6.69%), and “coenzyme transport and metabolism” (H, 22, 6.13%). The specific genes of the nine *V. atypica* strains are the primary contributors to the functions involved in “cell wall/membrane/envelope biogenesis,” “general function,” and “transcription.”

In addition, GO analysis was performed to characterize the genetic functions of *V. atypica* and obtain a deep understanding of the function catalogs of this species. Based on the genetic functions of the orthologous clusters, these genes were categorized into biological process, cellular component, and molecular function (Figures 3B,D). An enrichment analysis of biological processes revealed that high proportions of the core and the accessory genes contributed to the functions involved in “cellular process” (442, 58.01%), “metabolic process” (432, 56.69%), “cellular metabolic process” (396, 51.97%), “organic substance metabolic process” (392, 51.44%), “primary metabolic process” (354, 46.46%), “nitrogen compound metabolic process” (350, 45.93%), “biosynthetic process” (297, 38.98%), and “small molecule metabolic process” (203, 26.64%) (Figure 3B). The majority of the genes assigned to cellular components was grouped into “cell” (600, 78.74%), “intracellular” (368, 48.29%), “membrane” (368, 48.29%), “cell periphery” (356, 46.72%), and “plasma membrane” (324, 42.52%) (Figure 3C). An enrichment analysis of molecular function showed that many genes were grouped into the functions involved in “catalytic activity” (334, 43.83%), “binding” (150, 19.69%), “hydrolase activity” (118, 15.49%), and “transferase activity” (101, 13.25%) (Figure 3D).

Unlike the previous studies that focused on the pan-genome of *Aeromonas* and *Shewanella*, the present study found that the distribution of the functional categories of *V. atypica* was different from that of *Aeromonas* and *Shewanella*. The pan-genome analysis of the *Aeromonas* strains showed that several COG categories had a higher percentage in the accessory clusters than in the other clusters but were less represented in the core clusters, such as “energy production and conversion,” “amino acid transport and metabolism,” “carbohydrate transport and metabolism,” and “transcription” (Zhong et al., 2019). By comparison, the pan-genome analysis of 24 *Shewanella* strains showed that the functional traits affiliated with “plasma membrane” had a higher percentage in the specific clusters than the other functional traits but were less represented in the core and the accessory clusters (Zhong et al., 2018). In the present study, the functional traits of the *V. atypica* strains had a higher percentage in the core clusters than in the accessory and the specific clusters (Figures 3B,D), suggesting that the primary genetic functions of the *V. atypica* strains depended on its core genome of *V. atypica*, while the differences between the accessory and the specific clusters contributed to the other metabolism functions of *V. atypica*.

### Metabolic Pathways Annotated by KEGG Server for *V. atypica* Strains

To obtain a deeper understanding of the metabolic pathways of the *V. atypica* strains, we annotated the proteins of the nine *V. atypica* strains against KEGG database. The outputs of the KEGG Automatic Annotation Server (KAAS) server for the nine *V. atypica* strains are summarized in Supplementary Table S1. The metabolic pathways detected in the nine *V. atypica* strains were mainly involved in primary metabolic synthesis and secondary metabolic synthesis.

Firstly, a large proportion of the proteins was involved in the metabolic pathways of ribosome (4.57–4.8%), ABC transporters (3.78–4.37%), porphyrin and chlorophyll metabolism (3–3.3%), purine metabolism (2.87–2.97%), pyrimidine metabolism (2.42–2.56%), pyruvate metabolism (2.37–2.53%), cysteine and methionine metabolism (2.28–2.41%), two-component system (2.12–2.56%), aminoacyl-tRNA biosynthesis (2.24–2.32%), quorum sensing (2.17–2.4%), carbon fixation pathways in prokaryotes (1.85–2.08%), phenylalanine, tyrosine, and tryptophan biosynthesis (1.7–1.8%), and lipopolysaccharide biosynthesis (1.71–1.79%) (Supplementary Table S1). Notably, the number of genes detected in these pathways varied in the nine *V. atypica* strains, and several proteins were annotated to the KEGG pathways of antibiotic resistance, including vancomycin resistance and platinum drug resistance (Supplementary Table S1). Vancomycin resistance in genus *Veillonella* has already been reported (Al-Otaibi and Al-Mohizea, 2014; Carlier, 2015), but our new finding indicated that the functional information of the nine *V. atypica* strains was involved in the metabolic pathway of vancomycin, which is summarized in Supplementary Tables S2, S3. Our results reinforced the results of previous studies associated with vancomycin in *V. atypica* and provided valuable information from the perspective of bioinformatics and pan-genome, which is helpful for researchers who are interested in the *V. atypica* strains to conduct more in-depth research.

Secondly, based on the annotations of the proteins for the nine *V. atypica* strains, we constructed the pathway maps and the modules for the nine *V. atypica* strains. These pathway maps and modules are summarized in Supplementary Tables S2, S3. Among the integrated metabolic pathways, a large proportion of the proteins was involved in metabolic pathways, ranging from 19.08 to 19.41%, for the nine *V. atypica* strains (Supplementary Table S2). The top pathways annotated with maximum KEGG orthology hits were biosynthesis of secondary metabolites (8.17–8.35%), biosynthesis of antibiotics (5.62–5.85%), microbial metabolism in diverse environments (4.36–4.5%), and biosynthesis of amino acids (4.12–4.27%) (Supplementary Table S2) in the nine *V. atypica* strains. Combined with the information provided in Supplementary Table S1, we found that the KEGG pathways were involved in the biosynthesis of antibiotics, including novobiocin biosynthesis, streptomycin biosynthesis, carbapenem biosynthesis, prodigiosin biosynthesis, acarbose and validamycin biosynthesis, penicillin and cephalosporin biosynthesis, biosynthesis of ansamycins, and biosynthesis of vancomycin group antibiotics. Additionally, 138 KEGG modules were identified in the nine *V. atypica* strains (Supplementary Table S3). The distributions of the number of genes identified in these pathways and modules were almost the same in the nine *V. atypica* strains.

Overall, an investigation of the metabolic pathways of the nine *V. atypica* strains will expand our knowledge of these processes and also aid in the transformation of the metabolic pathways of several substrates. Besides that, it should be noted that the prediction of functional traits and metabolic pathways of the *V. atypica* strains in this present study depended on their genomes, whereas in the actual natural situation, microorganisms exist in the form of a microbial community. Following previous studies (Zhang et al., 2018; Zhong et al., 2019), to further profile and confirm the functional composition of the *V. atypica* strain, it is necessary to conduct a deeper analysis based on the metagenomic data and/or (meta-)transcriptomic data of the microbial communities containing the *V. atypica* strains. Moreover, the functions, especially the metal reducing mechanism, of the *V. atypica* strains need to be verified *via* wet experiments in further studies.

### CRISPR–Cas Systems for *V. atypica* Strains

To efficiently edit the genetic elements of the metabolic pathways of *V. atypica*, we identified the CRISPR array and the *Cas* proteins of CRISPR–Cas systems for the nine *V. atypica* strains. Firstly, the distribution patterns of the number of CRISPR arrays were different among the nine *V. atypica* strains (Table 2). *V. atypica* ACS-049-V-Sch6 harbored the lowest number of CRISRP arrays (two), whereas *V. atypica* ACS-134-V-Col7a harbored the highest number of CRISPR arrays (12) (Table 2). The CRISRP arrays are DNA segments captured by prokaryotic bacteria from invading viruses and suited as virus recognition keys. The CRISPR arrays consist of a number of spacers which, in a CRISPR array of a prokaryotic bacteria, are maximized for protection against viral attacks (Martynov et al., 2017). Owing to the repetitive nature of the repeating sequences, the CRISPR arrays have been proven difficult to generate. Variations in the number of CRISPR arrays among the nine *V. atypica* strains suggested that the ability of these nine *V. atypica* strains to accept foreign DNA was different. Secondly, the *Cas* proteins were detected in *V. atypica* ACS-049-V-Sch6 (two *Cas* proteins), *V. atypica* ACS-134-V-Col7a (four *Cas* proteins), *V. atypica* KON (three *Cas* proteins), *V. atypica* OK5 (one *Cas* protein), *V. atypica* KON ATCC 17744 (three *Cas* proteins), *V. atypica* KHUD V1 (three *Cas* proteins), and *V. atypica* NCTC11830 (one *Cas* protein) (Table 2). By contrast, the *Cas* proteins were not identified in *V. atypica* CMW7756B and *V. atypica* AF36-15BH, probably because the genomes of these two strains were not complete. The *Cas* proteins are known to use the genetic information stored in the CRISPR arrays to initiate a host immune response against mobile genetic elements that carry a homologous sequence to achieve gene editing (Westra et al., 2016). The variation in the number of *cas* genes among these nine *V. atypica* strains likely resulted from rapid evolution and extensive horizontal gene transfer (Westra et al., 2016), which revealed the variation of the CRISPR–Cas system among the *V. atypica* strains and suggested that it is important to obtain a deeper understanding of the genetic background of the *V. atypica* strains to achieve gene editing effectively. Thirdly, we observed that these seven *V. atypica* strains almost harbored CAS-Type IIIA, and we detected CAS-Type IIA, CAS-Type IIC, and CAS-Type IIID in the *V. atypica* strains (Table 2). Taken together, these results suggested that various and diverse *Cas* proteins can be selected to edit the genetic elements of the *V. atypica* strains.

**TABLE 2:** CRISPR arrays and *Cas* proteins detected in the CRISPR–Cas systems of the nine *Veillonella atypica* strains.

Strain	No. CRISPR array	No. Cas genes	Cas-type/subtype
*V. atypica* ACS-049-V-Sch6	2	2	CAS-TypeIIIA (Casl0_0_IIIA,Csm2_0_IIIA, Csm3_0_IIIA, Csm4_0_IIIA, Csm5_0_IIIA, Csm6_0_I CAS_putative (Casl_0_I-II-III, Cas2_0_I-II-III-V, Cas6_0_I-III)
*V. atypica* ACS-134-V-Col7a	12	4	CAS_putative (Casl_0_I-II-III)
			CAS_putative (Cas6_0_I-III)
			CAS-TypeIIID (Cas10_0_III, Csm3_0IIIAD, Csm3_0_IIID, Csm3_l_IIID)
			CAS-TypeIIA (Cas9_0_II, Casl_0_II, Cas2_0_I-II-III, Csn2_0_IIA)
*V. atypica* KON	10	3	CAS_putative (Cas3_0_I)
			CAS-TypeIIIA (Cas10_0_IIIA, Csm2_0IIIA, Csm3_0IIIA, Csm4_0IIIA, Csm5_0_IIIA, Csm6_0_ CAS_putative (Casl_0_I-II-III, Cas2_0_I-II-III-V, Cas6_0_I-III)
*V. atypica* CMW7756B	3	Not detected	
*V. atypica* OK5	10	1	CAS-TypeIIIA (Cas2_0_I-II-III-V, Casl_0_I-II-III, Csm6_0_IIIA, Cas6_0_I-III, Csm5_0IIIA, Csm4_0_IIIA, Csm3_0_IIIA, Csm2_0_IIIA, Cas10_0IIIA)
*V. atypica* KON ATCC 17744	8	3	CAS-TypeIIIA (Cas10_0_IIIA, Csm2_0IIIA, Csm3_0IIIA, Csm4_0IIIA, Csm5_0_IIIA, Csm6_0_
			CAS_putative (Cas10_0_I-II-III, Cas2_0_I-II-III-V,Cas6_0_I-III) CAS_putative (Cas3_0_I)
*V. atypica* AF36-15BH	4	Not detected	
*V. atypica* KHUD VI	5	3	CAS-TypeIIA (Csn2_0_IIA)
			CAS_putative (Cas2_0_I-II-III)
			CAS-TypeIIC (Cas9_0_II, Casl_0_II, Csn2_0IIA)
*V. a*/*yp*z*ca* NCTC11830	7	1	CAS-TypeIIIA (Cas2_0_I-II-III-V, Casl_0_I-II-III, Csm6_0_IIIIA, Cas6_0_I-III, Csm5_0IIIA, Csm4_0_IIIA, Csm3_0_IIIA, Csm2_0_IIIA, Cas10_0_IIIA)

## Conclusion

In conclusion, we performed a comparative analysis on the pan-genome of species *V. atypica* on the basis of complete and draft genomes of the *V. atypica* strains in this study. The results demonstrated that *V. atypica* exhibited an open pan-genome structure. A phylogenetic analysis revealed that *V. atypica* OK5 may not be the suitable representative strain for *V. atypica*. Moreover, the results suggested that another strain or additional strains are needed to be selected for sequencing and model construction for *Veillonella* genus in future studies. The functional annotations of the nine *V. atypica* strains were profiled against the CAZyme, COG, and GO databases. Our results demonstrated that the differences in CAZymes among the nine *V. atypica* strains were attributed to the CAZyme component derived from the accessory clusters. Additionally, the core clusters were more highly represented in the COG and the GO annotations than the accessory clusters and the specific clusters, suggesting that the primary genetic functions of the *V. atypica* strains depend on the core genome of *V. atypica*. By contrast, the differences in the accessory clusters and the specific clusters contributed to the other metabolism functions of *V. atypica*. Moreover, several proteins were annotated to the KEGG pathways involved in antibiotic resistance and biosynthesis of secondary metabolites, demonstrating that the *V. atypica* strains harbored vancomycin resistance. Besides that, four main *Cas* proteins of the CRISPR–Cas systems for the *V. atypica* strains, namely, CAS-Type IIIA, CAS-Type IIA, CAS-Type IIC, and CAS-Type IIID, were harbored in the *V. atypica* strains. These various *Cas* proteins can be used to edit the genetic elements of the *V. atypica* strains. The genetic diversity and the functional traits of *V. atypica* strains were together uncovered using their genomic information in this present study. The present study found considerable differences among the *V. atypica* strains. Hence, the results suggested that we should have a deep understanding of the genetic background of the *V. atypica* strains before we realize their biological potential, including their probiotic potential for humans, clarification of the metabolic pathways associated with secondary metabolic synthesis, and potential application as selenium-respiring bacterium with an important role in the selenium cycle. Nevertheless, our pilot comparative study was on the basis of one complete genome (*V. atypica* OK5) and eight draft genomes (the others) of the *V. atypica* strains, and the limitations of the genetic datasets might have possibly affected the revelation of the real comparative analysis results of *V. atypica*, which suggests and requires researchers to isolate more *V. atypica* strains from more ecological niches, especially the *V. atypica* strains isolated from the gut, in future studies. Additional isolated *V. atypica* strains and their corresponding complete genomes should be used in the comparative analysis of *V. atypica*, and more attention should be paid to investigate the pan-genome analysis of *V. atypica* to optimize the potential applications of this bacterium in future studies. It is important to note that our study is a pilot study, and we focused on the genetic diversity and the functional characteristics of the *V. atypica* strains. This study provides an in-depth understanding of the genetic diversity, functional composition, and metabolic pathways of the *V. atypica* strains and profiles their CRISPR–Cas systems on the basis of the genomic information of the *V. atypica* strains. The results provide insights into the biosynthetic engineering of *V. atypica*.

## Materials and Methods

### Collection of Genomic Datasets of *V. atypica*

To date, nine strains of *V. atypica* have been sequenced, and their genomic datasets have been uploaded to the genome database of NCBI. The nine strains of *V. atypica* are *V. atypica* KON, *V. atypica* CMW7756B, *V. atypica* ACS-049-V-Sch6, *V. atypica* ACS-134-V-Col7a, *V. atypica* KON ATCC 17744, *V. atypica* NCTC11830, *V. atypica* AF36-15BH, *V. atypica* KHUD_V1, and *V. atypica* OK5. The genome and the protein sequences of these nine *V. atypica* strains were downloaded from the GenBank database of NCBI for comparative genomics analysis (Table 1). Among these nine *V. atypica* strains, according to the information from NCBI BioSample and LPSN, *V. atypica* KON, *V. atypica* KON ATCC 17744, and *V. atypica* NCTC11830 are defined as the same type of strain of *V. atypica*.

### Identification of the Protein Orthologs of *V. atypica*

To obtain a better understanding of genetics knowledge on *V. atypica* and the evolutionary relationship of the *V. atypica* strains, a systematic comparative genomic analysis of the *V. atypica* strains was conducted. The protein orthologs of *V. atypica* were identified using OrthMCL (version: 2.0.9) (Li et al., 2003), with an *e*-value < 1e-5 and an inflation parameter of 1.5 (Zhong et al., 2018). The protein homologous clusters were clustered into core, accessory, and specific groups. The core group represented the genes or the proteins shared by all nine *V. atypica* strains. The accessory group comprised the genes or the proteins shared by at least two strains but not by all nine *V. atypica* strains. The remaining genes or proteins occurring only in one *V. atypica* strain were cataloged into the specific group (strain-specific genes or proteins). In this present study, the clustering result of the proteins yielded 2,065 homologous clusters, 1,468 of which were identified as single-copy protein families.

### Construction of the Phylogenetic Tree of *V. atypica*

To obtain the evolutionary relationship of the nine *V. atypica* strains, three phylogenetic trees for these nine strains were constructed. The 16S rRNA sequences were extracted from the genomic sequences by using Barrnap,^2^ and then a phylogenetic tree on the basis of the 16S rRNA sequences was built with MEGA software (version: 7.0.26) (Tamura et al., 2011). The other phylogenetic trees were built with different strategies. Specifically, the protein sequences of these single-copy protein families were concatenated and aligned using MUSCLE (version 3.8.31) (Edgar, 2004), with default settings. The results of multiple protein sequence alignment were eliminated using Gblocks (version 0.91b) (Talavera and Castresana, 2007) to remove the regions that were divergent, misaligned, or with a larger number of gaps. Then, the phylogenetic tree was constructed in Phylip (version 3.696) (Jd, 1999) by using the maximum likelihood algorithm with 100 bootstrap iterations. Additionally, a pan-genome tree was constructed based on the absence or the presence of each homologous protein using the Manhattan distance to evaluate the evolutionary relationships among the nine *V. atypica* strains (Zhong et al., 2018, 2019). The phylogenetic trees were generated and visualized using MEGA software (version 7.0.26).

### Functional Annotations

To gain more insights into the functional characteristics of *V. atypica*, a total of 2,065 homologous proteins were assigned against the CAZyme, COG function, and eggNOG annotation databases. Specifically, we annotated the protein sequences of 2,065 homologous clusters against CAZy database, which was downloaded from dbCAN (Lombard et al., 2014),^3^ to explore the functional composition of *V. atypica* related to carbohydrate metabolism. According to the manual of dbCAN CAZyme annotation, the protein sequences were annotated by running hmmscan of HMMER (version: 3.1b1) (Cantarel et al., 2008; Mäkelä et al., 2018), with default settings, and the annotated results were summarized into GT, GH, carbohydrate-binding module, auxiliary activity, polysaccharide lyase, S-layer homology, and CE. The protein sequences of 2,065 homologous clusters were annotated to COG proteins (Tatusov et al., 2003), which were downloaded from NCBI,^4^ by using local BLASTP with a cutoff *E*-value of 1e-4. The top one of the annotation results was selected as the best annotation for each homologous cluster, and then it was assigned to 25 functional categories. The protein sequences of 2,065 homologous clusters were aligned to the eggNOG database by using the eggnog-mapper (Huerta-Cepas et al., 2015). GO annotations were summarized for the core, accessory, and specific groups based on the GO annotations of 2,065 homologous clusters, respectively. The results of the GO annotations of the core, accessory, and specific groups were divided into three groups, including biological process, molecular function, and cellular component, and then visualized using Web Gene Ontology Annotation Plotting (version 2.0) (Ye et al., 2018).

### KEGG Pathway Analyses

To obtain a comprehension of functional and metabolic interactions in the nine *V. atypica* strains, their proteins were submitted to the KAAS^5^ to identify the metabolic pathways (Moriya et al., 2007). When using the server, BLAST was chosen as the search program, and bi-directional best hit was selected as the preferred assignment method. Additionally, the references of default organisms for prokaryotes were chosen to obtain KEGG annotations.

### Prediction of CRISPR–Cas Systems for *V. atypica* Strains

In general, the CRISPR–Cas systems are used for editing the genomes of diverse organisms. To obtain a better understanding of the CRISPR–Cas systems of the *V. atypica* strains, we conducted a comprehensive characterization of the CRISPR–Cas systems in the nine *V. atypica* strains. In this present study, the online tool CrisprCasFinder^6^ (Martinez-Fleites et al., 2006) was applied to detect the components of the CRISPR–Cas system, including the CRISPR arrays and the *cas* genes of the nine *V. atypica* strains. In using CrisprCasFinder, 100 bp was selected as the size threshold of the flanking regions for CRISPR array identification, and the CRISPR array contained truncated repeats.

## Data Availability Statement

The raw data supporting the conclusions of this article will be made available by the authors, without undue reservation, to any qualified researcher.

## Author Contributions

MH and YZ designed the study. MH and DW collected and downloaded the data. MH, GL, and YZ analyzed the data. MH, GL, YC, and YZ wrote the initial draft of the manuscript. All the authors revised the manuscript.

## Conflict of Interest

The authors declare that the research was conducted in the absence of any commercial or financial relationships that could be construed as a potential conflict of interest.

## Footnotes

1 https://www.bacterio.net/

2 https://github.com/tseemann/barrnap

3 http://csbl.bmb.uga.edu/dbCAN/

4 https://www.ncbi.nlm.nih.gov/COG/

5 http://www.genome.jp/tools/kaas/

6 https://crisprcas.i2bc.paris-saclay.fr/CrisprCasFinder/Index
