# Supplementary material for: Comparative Genomics Uncovers the Genetic Diversity and Characters of Veillonella atypica and Provides Insights Into Its Potential Applications
Source: Front Microbiol. 2020 Jun 23;11:1219. doi: 10.3389/fmicb.2020.01219 (PMC7324755; doi:10.3389/fmicb.2020.01219)
Supplement: Supplementary file 4 [file Image_1.pdf]

Comparative Genomics Uncovers the Genetic Diversity and Characters of *Veillonella atypica* and provides Insights into its Potential Applications

Maozhen Han[1], Gang Liu[1], Yajun Chen[2], Dong Wang[2], Yan Zhang[2, *]

[1] School of Life Sciences, Anhui Medical University, Hefei, Anhui 230032, China
[2] School of Life Sciences, Hefei Normal University, Hefei, Anhui 230601, China
[*] Corresponding author. E-mail: zhangyanhfnu@gmail.com

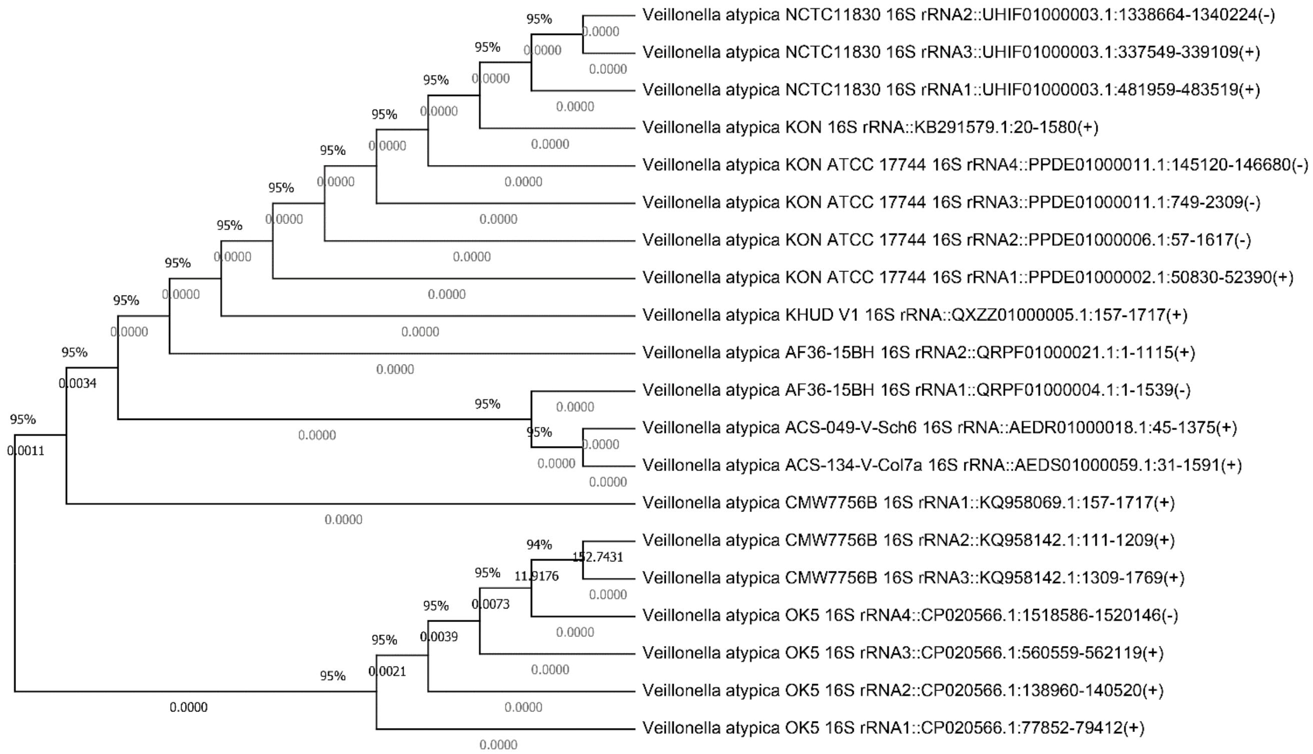

Supplementary Figure S1. The phylogenetic tree built based on 16S rRNA sequences of nine *V. atypica* strains. The 16S rRNA sequences were extracted from the genomic sequences of nine *V. atypica* strains by using Barrnap (https://github.com/tseemann/barrnap) and the phylogenetic tree was built with MEGA software with default parameters. The number of 16S rRNA gene copies ranged from 1 to 4. For example, one 16S rRNA gene copy was presented in *V. atypica* KON, four 16S rRNA gene copies were presented in *V. atypica* KON ATCC 17744, and three 16S rRNA gene copies were presented in *V. atypica* NCTC11830. The result of the phylogenetic tree showed that *V. atypica* KON, *V. atypica* NCTC11830, and *V. atypica* KON ATCC 17744 are in the same cluster and revealed that these strains are same strains. However, the results of similarity analysis among the genomes of *V. atypica* KON, *V. atypica* NCTC11830, and *V. atypica* KON ATCC 17744 showed that the symmetric identify between *V. atypica* KON ATCC 17744 and *V. atypica* KON is 98.91%, while the symmetric identify between *V. atypica* KON ATCC 17744 and *V. atypica* NCTC11830 is 97.51%. These results suggested that *V. atypica* KON, *V. atypica* KON ATCC 17744, and *V. atypica* NCTC11830 are different substrains of *V. atypica* KON.
